# Evaluation of Health Hazard Due to Emission of Volatile Organic Compounds from Various Processing Units of Wastewater Treatment Plant

**DOI:** 10.3390/ijerph16101712

**Published:** 2019-05-16

**Authors:** Hubert Byliński, Jacek Gębicki, Jacek Namieśnik

**Affiliations:** 1 Department of Analytical Chemistry, Faculty of Chemistry, Gdańsk University of Technology, Narutowicza 11/12 Street, 80-233 Gdańsk, Poland; jacek.namiesnik@pg.edu.pl; 2 Department of Process Engineering and Chemical Technology, Chemical Faculty, Gdańsk University of Technology, Narutowicza 11/12 Street, 80-233 Gdańsk, Poland; jacek.gebicki@pg.edu.pl

**Keywords:** wastewater treatment plant, odour concentration, health risk, odour emission, odour

## Abstract

The paper describes an attempt at health risk assessment and odour concentration determination in the most important units of a wastewater treatment plant. The cancer risk (CR) and hazard index (HI) parameters in selected measurement locations were calculated based on the results of chromatographic analyses (GCxGC-TOF-MS) and the United States Environmental Protection Agency (US EPA) guidelines. No exceedance of the CR and HI acceptable levels was observed for identified and quantitatively determined compounds from the VOCs group. The acceptable level was exceeded for the summary HI parameter. Following a classification of the International Agency for Research on Cancer (IARC), it was noticed that the highest hazard was connected to the presence of formaldehyde belonging to group 1—the compounds regarded as carcinogenic. Based on the olfactometric analyses, it was estimated that the highest odour concentration, 37.2 ou/m^3^, occurred at the solid waste composting piles. It was also revealed that an increase in odour concentration corresponded to a higher health risk for employees of the wastewater treatment plant, due to exposure to volatile odorous compounds. Accordingly, this method of odour measurement can be a fast indicator describing health risk level.

## 1. Introduction

Municipal wastewater treatment plants (WWTPs), despite their beneficial contribution to environmental protection, are recognised as one of the potential emission sources of atmospheric pollutants [1,2,3,4]. The main group of compounds emitted from the treatment plants are volatile organic compounds [5,6]. Their presence in ambient air can result in the discomfort of the plant’s employees as well as the residents of neighbouring areas, even when those compounds are present at very low concentration levels. Moreover, some of these compounds can cause a number of psychosomatic symptoms such as anxiety, stress, headache and dizziness, nausea, loss of consciousness. The odorous substances characterised by low olfactory threshold can have significant impact on discomfort associated with the perception of unpleasant odour of different intensity and hedonic tone [7,8].

Wastewater directed to particular treatment stages can differ in chemical composition [9]. That is why the type and amount of volatile odorous compounds generated at any given wastewater and sludge treatment stage can differ substantially. At the beginning, the wastewater stream is usually subjected to mechanical treatment where solid waste, grease and small mineral fractions are removed. These operations frequently produce many volatile odorous compounds. A solid fraction isolated during the mechanical treatment is rich in various precursors of odorous compounds, so it is important to select proper methods of its further processing (stabilisation, composting or incineration) [10]. Lower levels of odorants emission are observed in case of separation of wastewater from sludge in the preliminary settling tanks. It is due to the fact that at this stage no operations of mechanical separation of solid fractions are carried out (separation occurs via sedimentation and flotation) [11]. A number of metabolic processes take place in the biological reactors; they proceed in aerobic and anaerobic conditions, in presence of microorganisms. These processes are often accompanied by a release of the odorous compounds. Their emission can be limited by the introduction of bacterial strains, which neutralise selected groups of odorous compounds [12]. However, it is necessary to provide and maintain suitable conditions, ensuring efficiency of biological removal of nitrogen and phosphorus. After leaving the biological reactors, the stream of wastewater and biological sludge enters the secondary settling tanks where separation occurs. At this stage, wastewater is usually lean in the odorous compounds. The substances, which can contribute to odour include: 2,4,6-trichloroanisole, geosmin, 2-methyloisoborneol [13].

The operations connected to processing and neutralisation of sludge are characterised by different levels of volatile odorous compounds emission [14]. One of the most frequently applied solutions, especially in bigger treatment plants, is methane fermentation with biogas recovery [15]. Due to its anaerobic character, this process generates significant emission of the odorous compounds, which is present in the post-fermentation sludge as well as in recovered biogas. The fermentation processes are usually carried out in closed chambers, which limits emission of odorants to the environment. The post-fermentation sludge can be rich in the odorous compounds, so it must be subjected to additional operations, such as dewatering, drying, biological stabilisation, before further management, for instance in agriculture [16]. Thermal processing of sludge gains has had increasing popularity as it allows for the transformation of organic compounds into stable forms, which are not hazardous to the environment [12].

Thus, it seems justified to conduct the research, which would describe the influence of selected volatile organic compounds released to ambient air on human health and related long-term hazards.

The magnitude of exposure risk for a wastewater treatment plant’s employees depends on many factors, such as the size of the plant, amount and character of sewage entering the plant or employed technological solutions, including proper operation of the plant [17,18]. One of the most common tools used to evaluate the impact of a hazard on human health is risk assessment [19]. This technique allows for the identification of specific environmental problems and development of appropriate management strategies related to some hazardous chemicals, which can have a negative effect on both abiotic and biotic parts of the environment. Qualitative analysis of human health risks assumes that chemicals are distinguished between two categories: carcinogenic and non-carcinogenic, based on their abilities to induce some adverse health effects depending on the threshold level. In the literature, there are many examples of application of this approach to evaluate health risk of VOCs emitted from landfills and composting plants [17,20,21].

One of the main international organisations dealing with the classification of carcinogenic chemical factors is the International Agency for Research on Cancer (IARC). Its aim is the investigation of the contribution and human health risk-related properties of various factors, which could be present at the workplace and in the natural environment. The results of activity of particular working groups of the IARC are successive volumes of the contribution IARC *Monographs on the Evaluation of Carcinogenic Risks to Humans*, which contain the information on human health risk imposed by particular chemical compounds. Five categories of carcinogenic factors have been determined based on a number of criteria adopted by the IARC:

- Group 1—factor or set of factors carcinogenic to people;
- Group 2A—factor or set of factors, which is probably carcinogenic to people;
- Group 2B—factor or set of factors, which is presumably carcinogenic to people;
- Group 3—factor or set of factors, which cannot be classified as carcinogenic to people;
- Group 4—factor or set of factors, which are probably non-carcinogenic to people.

Despite numerous legal regulations introduced in many countries all over the world by the organisations such as the IARC, it is not a common practice to evaluate health risk connected with the volatile organic compounds generated during wastewater treatment plant operation. According to the authors, such evaluation should additionally take into account the determination of odour nuisance magnitude connected with sewage and sludge processing. Odour concentration can be supporting information about gaseous mixtures of VOCs. The presence of an unpleasant smell in any place is a form of indication, which can inform us about some human health risks [22]. Integrated risk assessment on the olfactory characterisation and human health effects of volatile compounds emissions from different processing units in WWTP have not been well studied. The investigations presented below concern the health hazard of wastewater treatment plant workers exposed to VOCs during working hours.

Based on the guidelines proposed by the US Environmental Protection Agency [23] and the classification of potentially carcinogenic compounds implemented by the IARC, this paper presents health risk parameters of cancer risk (CR) and hazard index (HI) determined for 5 selected compounds, which have been found to impose carcinogenic risks, and for 24 selected compounds, which have been found to impose non-carcinogenic risks. The investigations were performed using the instrumental and sensory methods in 4 main processing units of a wastewater treatment plant during spring and summer seasons in two consecutive years: 2017 and 2018. Evaluation of the CR and HI parameters was conducted according to the methodology proposed by the US EPA and the classification introduced by the working groups of the IARC [19] for the compounds identified using the GCxGC-TOF-MS technique, comparing the obtained spectra with the NIST spectra library and with the spectra of reference substances. The aim of the investigation is the determination of the wastewater treatment plant workers health risk due to carcinogenic and non-carcinogenic compounds as well as the health hazard connected with odour nuisance. According to the authors, it is one of the first attempts at simultaneous evaluation of carcinogenic and non-carcinogenic risks associated with emission of volatile odorous compounds released at different stages of wastewater treatment plant operation and determination of odour concentration levels. To our best knowledge, this type of investigation has not been performed so far regarding simultaneous utilisation of instrumental and sensory tools for health risk evaluation associated with the operation of wastewater treatment plants.

## 2. Materials and Methods

### 2.1. Sampling Site and Air Sampling Methods

A municipal wastewater treatment plant, located in the northern part of Poland, was selected for investigation. This object is one of the northernmost treatment plants in Poland. It is located close to the seaside, which is a popular tourist attraction. This treatment plant is a mechanical–biological WWTP with anaerobic fermentation of sludge, conducted with biogas recovery and its utilisation in a cogeneration process. Solid waste after mechanical treatment, such as sand and screenings, as well as post-fermentation sludge are subjected to composting processes. Table 1 presents the most important information concerning the amount of wastewater and sludge generated during the investigation period.

**Figure 1:**
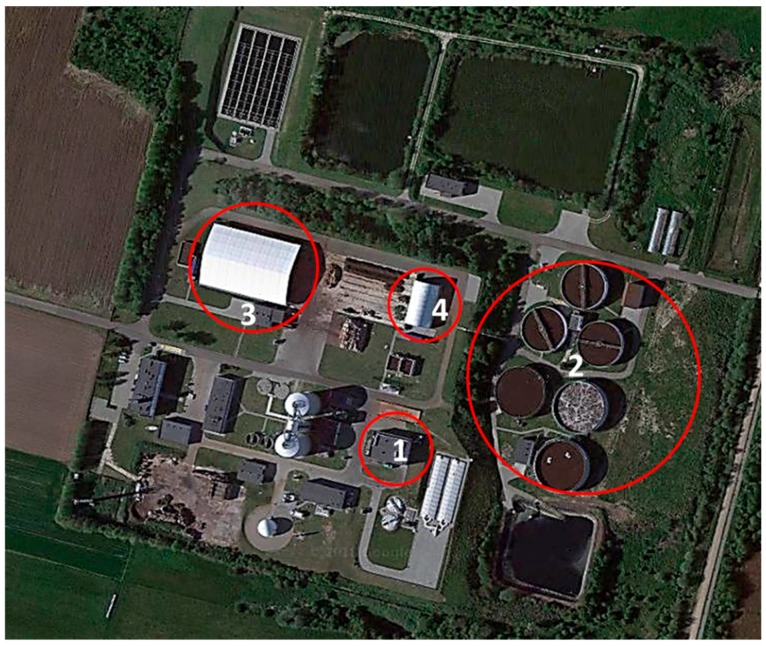
Measurement locations at WWTP; 1—hall of mechanical treatment (HMT), 2—biological reactors area (BRA), 3—sludge composting piles (SCP), 4—solid waste composting piles (SWCP).

Air samples from the WWTP units were collected in spring and summer of 2017 and 2018. The spring period was from mid-March until mid-June, whereas the summer period started in mid-June and finished in mid-September. To characterise volatile organic compounds in various processing units, the air samples were collected at four different functional locations of the WWTP, presented in Figure 1: hall of mechanical treatment (HMT), biological reactors area (BRA), sludge composting piles (SCP) and solid waste composting piles (SWCP) from mechanical treatment section. Based on the literature, at these places odorous VOCs can be most significant, from a health risk standpoint. Air sampling was performed between 08:00 and 12:00 or between 13:00 and 15:00, i.e., in working hours, to ensure that the samples represented the air the WWTP employees would have inhaled. However, air disturbances caused by the employees were avoided. From each measurement location, a total of 36 air samples were taken (3 samples during every week). Hence, 144 samples were taken for each sampling campaign, leading to a total of 288 samples in spring and summer of 2017 and 288 samples in spring and summer of 2018.

At each measurement location, volatile compounds were adsorbed on a glass tube containing poly(2,6-diphenyl-p-phenylene oxide) known by its trademark Tenax TA, using a Gas Sampling System (GSS, GERSTEL, Germany). Analytes adsorption on the sorbent bed is caused by flow of air containing measured compounds. In this way, it is possible to collect the substances present in gas medium, for example in ambient air. In order to purify the sorbent from impurities left over from the previous analysis, each sorbent tube was thermally desorbed at 280 °C for 3 h, using high purity nitrogen. During each measurement series, the air stream was passed through the glass tube at the volumetric flow rate 75 mL/min for 15 min. After sampling, the sorbent tubes were stored in a sealed package at about 4 °C, for no longer than 48 h.

### 2.2. Sensory Analysis

According to the European Standard (EN 13725:2003), dynamic olfactometry is the recommended method for determination of odour concentration. Application of a field olfactometer is advised in case of measurement of odour emission from diffusion sources or for periodical emission as well as low and fast-changing odour concentrations. Therefore, the *Nasal Ranger* field olfactometer was used in this research. This device allows for a sensory evaluation based on the proportion of odorous air and the air passed through a dedicated carbon filter attached to the olfactometer. Before the measurement series, a preliminary test of individual olfactory threshold, using aqueous solution of n-butanol (according to the standard procedure development by the St. Croix Sensory, Inc., Stillwater, MN, USA), was conducted. As a result, from a group of 25 volunteers, four panellists (2 females, 2 males, aged 26–30) were chosen to conduct field measurements. Each panellist, during the measurement series, conducted 3 evaluations in each location at 10-min intervals.

During the olfactometric analysis, “dilution to threshold ratio” (D/T) values, which show the ratio of the air stream that passed through the carbon filter (V_clean_) to the odour-containing air stream (V_crude_), were measured. Based on D/T values, odour concentration was calculated according to the following Equations (1)–(4) [24]:

(1) $$Z_{YES}={\left(\frac{D}{T}\right)}_{YES}+1$$

(2) $$Z_{NO}={\left(\frac{D}{T}\right)}_{NO}+1$$

(3) $$Z_{ITE}=\sqrt{Z_{YES}\times Z_{NO}}$$

(4) $$c_{od}=\sqrt[{n}]{\overset{n}{\underset{0}{\prod }}Z_{ITE}}$$

where: Z_YES_—dilution corresponding to the D/T value at which the odour is first perceived; (D/T)_YES_—olfactometer disc position at which the odour is first perceived, directly succeeding the position at which it is yet imperceptible; Z_NO_—dilution corresponding to the D/T value at which the odour is not yet perceptible; (D/T)_NO_—olfactometer disc position at which the odour is imperceptible, directly preceding the position at which it can be perceived; Z_ITE_—individual olfactory threshold; c_od_—odour concentration (ou/m^3^).

### 2.3. Analytical Method

The air samples were analysed using a thermal desorption unit—two dimensional gas chromatograph (Agilent Technologies, Palo Alto, CA, USA) coupled with a time-of-flight mass spectrometer (LECO Corp., St. Joseph, MI, USA). The sampling tubes were thermally desorbed for 10 min at 300 °C with a flow of pure helium gas passing through, carrying the desorbed gases to a pre-concentration trap, thermally desorbed at 300 °C and then the gases were transferred to the GCxGC-TOF-MS. Two capillary columns were used for the analysis: the first—Equity 1 (30 m × 0.25 mm × 0.25 µm) from the Supelco (Bellefonte, PA, USA); the second column—SolGel-Wax (2 m × 0.1 mm × 0.1 µm) was purchased from the SGE Analytical Science (Austin, TX, USA). Separation of the sample components was performed using the following program for the primary oven: initial temperature 40 °C, kept for 1 min, ramped at 10 °C/min to 90 °C, ramped at 3 °C/min to 240 °C and held for 3 min; for the secondary oven: initial temperature 45 °C, kept for 1 min, ramped at 10 °C/min to 95 °C, ramped at 3 °C/min to 245 °C and held for 3 min. As a carrier gas, high purity hydrogen (N6.0 class) with the constant flow rate 1 mL/min was used. Temperature of the transfer line and the ion sources was 250 °C. The voltage of detector was set at 1600 V. The modulation time was 5 s. Ions in the m/z = 40–500 range were analysed. Data analysis from chromatographic separation was done using the algorithm for peak deconvolution implemented in the ChromaTOF software (LECO Corp., version 4.24, St. Joseph, MI, USA).

The first stage of instrumental investigations was qualitative analysis of the samples using the GCxGC-TOF-MS technique in order to identify the entire spectrum of the compounds present in ambient air at selected measurement sites. The obtained list of compounds was compared with the NIST spectra library and with the spectra of reference compounds in order to ensure unequivocal identification. In this way 24 compounds were selected for further studies. The second stage of instrumental measurements was quantitative analysis of the compounds identified before. It was conducted with TD-GCxGC-TOF-MS. Quantitative determination involved an external reference method [25]. The curve was constructed based on certified references of measured substances, just as in the case of the acquisition of the qualitative spectra at the compounds identification stage. The conditions of chromatographic measurements were identical at both the qualitative and quantitative analyses stages. The third stage was analysis of data from the RAIS and IRIS libraries [23] in order to determine for which of these 24 compounds it would be possible to calculate the CR and HI parameters, which describe whether a given compound can contribute to carcinogenic and non-carcinogenic risks. CR values could be determined for 5 compounds; for the remaining compounds it was possible to evaluate the HI parameter, which means that regardless of their concentration they did not contribute to an increase in the carcinogenic risk (the CR parameter).

### 2.4. Human Health Risk Assessment

The health risks imposed by VOCs inhaled by the plant’s staff were assessed according to the US Environmental Protection Agency [23]. The risk of carcinogenic and non-carcinogenic effects is affected by inhalation exposure to the odorous substances with toxicological parameters. The maximum exposure concentration for an individual volatile organic compound (EC_i_) was calculated according to the Equation (5):

(5) $$EC_{i}=\frac{c_{i}\times DET\times EF\times ED}{AT\times 365\times 24}$$

where: c_i_—emission concentration for each volatile organic compound (ug/m^3^) determined with GCxGC-TOF-MS system, DET—daily exposure time (6 hours/day in this study), EF—exposure frequency (350 days/year in this study), ED—exposure duration (35 years in this study) and AT—average time (25 years for non-carcinogenic risks and 70 years for carcinogenic risks). According to the US EPA guidelines, in the case of carcinogenic risk AT = lifetime and 70 years were accepted, similar to the other papers concerning carcinogenic risk in the vicinity of municipal plants; in the case of non–carcinogenic risk AT = exposure duration and 25 years were accepted similarly to the other papers. This time period was estimated as an average working time of the employee having contact with the installations such as wastewater treatment plants. However, these US EPA guidelines emphasise that in many cases, determination of the exposure duration can be troublesome because workers of the plant and residents of the area neighbouring the emission sources are exposed at varying levels and to different extents.

For carcinogenic effects, the health risk was calculated as a cancer risk (CR) according to the Equation (6):

(6) $$CR_{i}=EC_{i}\times IUR_{i}$$

where: IUR_i_—inhalation unit risk value ((ug/m^3^)^−1^). For non—carcinogenic effects, the health risk is expressed as the hazard index (HI), calculated based on the Equation (7):

(7) $$HI_{i}=\frac{EC_{i}}{RfC_{i}}$$

where: RfC—reference concentration (ug/m^3^). Both IUR and RfC values were taken from IRIS (Integrated Risk Information System) and RAIS (Risk Assessment Information System) databases [26,27].

Additionally, the chemical substances identified with the GCxGC-TOF-MS apparatus were classified according to the categories adopted by the IARC, presented in Section 1.

## 3. Results and Discussion

### 3.1. Volatile Organic Compounds Emission in WWTP

The volatile organic compounds, identified in selected locations at the wastewater treatment plant, represented different classes, mainly alcohols, aldehydes, ketones, esters, aromatic hydrocarbons and volatile organosulphur compounds. They are the substances frequently encountered in a vicinity of municipal plants, such as landfills or wastewater treatment plants [28,29]. Table 2 presents the percentage contribution of the most important compound classes in particular measurement locations in the conducted studies in 2017–2018. It is clear that the contribution of compound classes varies depending on the character of operations performed in a given location. Organosulphur compounds (21.1%—summer seasons, 22.9%—spring seasons) and aromatic hydrocarbons (20.8%—summer seasons, 19.7%—spring seasons) dominate in the hall of mechanical treatment. This observation can be explained by the fact that raw sewage, not subjected to any previous treatment, is exceptionally rich in these compounds. The sewage from households as well as industrial plants introduces a large amount of the aforementioned compounds [30]. Aliphatic hydrocarbons (24.8%—summer seasons, 23.1%—spring seasons) dominate in the air samples from the biological reactors area. They are characterised by much lower odour nuisance than aromatic hydrocarbons or organosulphur compounds, which is reflected in lower values of olfactory threshold that can be found in the literature [31]. The second major group in this location is ketones, the olfactory properties of which are diversified. The remaining two locations were dominated by organosulphur compounds (SCP: 29.1%—summer seasons, 29.9%—spring seasons, SWCP: 31.4%—summer seasons, 30.8%—spring seasons). Even at very low concentrations, these compounds reveal a strong odour. Hence, perceived odour strength was the highest in these two locations.

Table 3 gathers the main chemical compounds identified at the area of investigated wastewater treatment plant, together with their olfactory thresholds and information about odour profiles. Odour nuisance associated with the processes and the unit operations carried out in wastewater treatment plants results not only from the olfactory properties of particular compounds, but also from synergistic effects of the odorants. It significantly hinders evaluation of the influence of these compounds on biotic and abiotic environment. The chemical compounds identified in sewage treatment plants can originate from sewage, some of them can be produced during sewage transport to the plant or during sewage treatment in different conditions of temperature, humidity, pH or possible presence of biological material and chemicals. An important factor, influencing odorants generation, is also oxygen content during sewage and sludge treatment. Low oxygen content in the sewage inlet channels favours the production of a significant amount of organosulphur compounds and carbonyl compounds.

### 3.2. Human Health Risk Assessment

**Figure 2:**
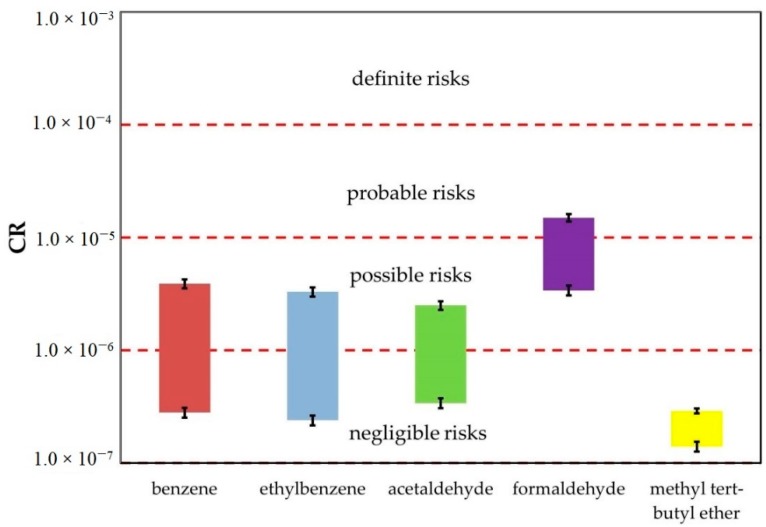
Ranges of cancer risk (CR) parameter determined based on investigations performed in both 2017 and 2018.

The CR values were calculated for 5 compounds: 2 aromatic hydrocarbons (benzene and ethylbenzene), 2 aldehydes (acetaldehyde and formaldehyde) and for methyl tert-butyl ether. The highest values were observed for formaldehyde. The US EPA acceptable CR values for individual substances is 1.0 × 10^−6^. Based on literature, the CR values larger than 1.0 × 10^−4^ are considered to be “definite risks”, the substances with CRs from 1.0 × 10^−4^ to 1.0 × 10^−5^— “probable risks”, the substances with CRs from 1.0 × 10^−5^ to 1.0 × 10^−6^— “possible risks” and the substances with CRs lower than 1.0 × 10^−6^ are considered to be “negligible risks” [20]. In our study, all CR values for formaldehyde exceeded the acceptable carcinogenic risk level (for both spring and summer). For methyl tert-butyl ether, the CR values were significantly smaller than the acceptable carcinogenic risk level. In all cases, the CR values presented in Figure 2 were smaller than 1.0E-04 and, based on the CR values for individual compounds, health risk can be considered not higher than “probable risks”, except for formaldehyde. Figure 2 presents the CR values obtained in the research during spring–summer of 2017–2018 with classification of the CR values according to previously cited literature.

**Figure 3:**
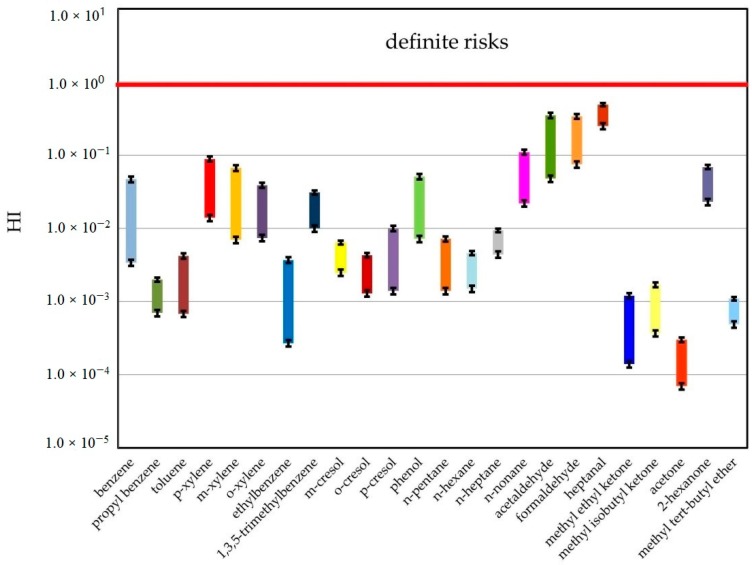
Ranges of hazard index (HI) parameter determined based on investigations performed in both 2017 and 2018.

All HI values were not higher than the acceptable risk level for individual compound (HI = 1) [32], which was presented in Figure 3. A comparison of the HI parameter reveals that, regardless of the measurement location, the highest values were obtained for three aldehydes: acetaldehyde, formaldehyde and heptanal In some papers on health risk assessment, the HI parameter above 0.5 indicates “probable risk” [16].

Comparing our results with the ones obtained by other authors, Mustafa et al. [20] performed human health risk assessment related to the emission of volatile organic compounds generated during the unit operations connected with organic fraction degradation in one of the composting plants in China. The investigations were conducted in the winter and summer seasons, in order to evaluate differences in potential risk associated with the VOCs emission in different atmospheric conditions. The results revealed that the CR and HI parameters were below the acceptable limit (CR < 1.0 × 10^−4^ and HI < 1), except for those recorded for naphthalene in a biofilter (CR = 1.1 × 10^−4^, HI = 3.07 during the summer season). Marti et al. [33] also carried out a human health risk assessment, based on the CR and HI parameters, for one of the landfills in Spain. In all cases, obtained values were within the acceptable limit. Durmusoglu et al. [19] conducted a health risk assessment with respect to aromatic compounds emission (benzene, toluene, ethylbenzene and xylenes) from a landfill in Turkey. Also in this case, the CR and HI parameters did not exceed the acceptable limits, which led the authors to a conclusion that the emission of the compounds from the BTEX group did not impose a health risk to the landfill’s employees and to the residents of the neighbouring area. Table 4 presents a comparison of the CR and HI parameters obtained in this study with the results of other studies associated with emission of volatile compounds from various municipal facilities.

#### 3.2.1. Comparison of Average Concentration of Particular Chemical Compounds with the Acceptable Concentrations Averaged over One Year Period

Based on the information contained in Table 5, which compares average concentrations during the entire measurement period for identified and quantitatively determined compounds belonging to the groups of carcinogenic and non-carcinogenic substances according to the US EPA with the acceptable concentration levels averaged over one calendar year (according to the Polish regulations), it can be noticed that the compounds from the carcinogenic group, such as formaldehyde and acetaldehyde, reveal possible a health risk (although average concentrations are higher than the acceptable level but the averaging period was limited only to the spring and summer of 2017 and 2018, which constitutes ca. 7% of the entire annual contribution). It was also observed that in the the entire measurement period, the compounds from the non-carcinogenic group according to the US EPA, including toluene, m-xylene, p-xylene, o-cresol, m-cresol, p-cresol, phenol, methyl isobutyl ketone, were characterised by average concentrations higher than the acceptable levels, regarding the same time span. Such a situation enforces the critical approach to the CR and HI parameters obtained for given chemical compounds. Moreover, a reliable health risk assessment of the wastewater treatment plant workers requires continuous monitoring of ambient air regarding particular chemical compounds emitted during sewage treatment, with special emphasis on variable emission levels of volatile organic compounds, depending on the season of the year as well as on such parameters as temperature, solar radiation or relative humidity. Continuous monitoring generates additional cost and quantitative determination of a big number of carcinogenic and non-carcinogenic compounds according to the US EPA, IARC or other international organisation guidelines is laborious. Thus, it seems that the summary CR and HI parameters would be better indicators than the CR and HI parameters determined for individual chemical compounds. It requires application of holistic analysis, which is provided by the olfactometric techniques. It seems that correlation of the results obtained with instrumental techniques with the olfactometric techniques results would allow for a fast evaluation of health risk level.

Analysing particular compounds with respect to a given classification elaborated by the IARC working group, it can be found that not all carcinogenic compounds according to the US EPA are also carcinogenic according to the IARC. Benzene and formaldehyde belong to the group 1—carcinogenic compunds, ethylbenzene and acetaldehyde are in the group 2B—compounds presumably carcinogenic to people, whereas methyl tert-butyl ether is in the group 3—compound not classified as carcinogenic to people. It should be noted that, apart from 5 compounds included in Figure 2, methyl isobutyl ketone also belongs to the group 2B (presumably carcinogenic to people) according to the IARC. Remaining identified compounds are classified into the group 3 or are not included in the IARC documents. This situation implies careful usage of the CR and HI parameters for particular chemical compounds. What is required is the knowledge not only of national acceptable levels but also of international regulations and guidelines concerning the health hazard of the workers exposed to the entire spectrum of compounds, including carcinogenic and non-carcinogenic ones.

### 3.3. Odour Concentration and Total Carcinogenic and Non-Carcinogenic Risk

The information presented in Section 3.2 and Section 3.2.1 made the authors take a closer look at the problem of health risk of the workers exposed to inhalation of carcinogenic, non-carcinogenic as well as odour-nuisance generating compounds, which contribute to discomfort at wastewater treatment plants.

**Figure 4:**
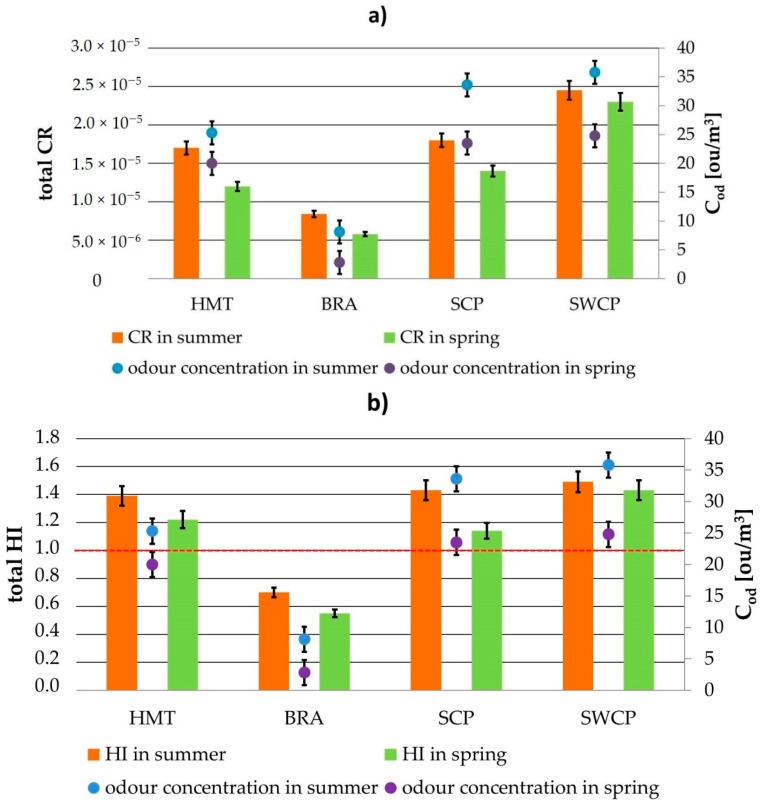
Total carcinogenic (**a**) and non-carcinogenic (**b**) risk in comparison to odour concentration values based on investigations performed in both 2017 and 2018.

Conducted olfactory measurements made it possible to assess the odour concentrations in given locations, both during spring and summer seasons. These data together with summary values of the CR and HI parameters, determined separately for each measurement location and period, are presented in Figure 4a,b. The summary values of the CR and HI parameters were calculated as a sum of these parameters obtained for particular compounds. Utilisation of the summary values seems more convenient due to the fact that the people present in the given locations are exposed to simultaneous impact of volatile odorous compounds mixture, not to its particular components separately.

Comparing the summary CR and HI values with the acceptable levels, it can be noticed that in the case of the carcinogenic risk, the summary CR values in each measurement location do not exceed the acceptable level (1.0 × 10^−4^); for the summary HI values, HI < 1 only in case of the biological reactors area. In the remaining three locations, the acceptable level is exceeded to the value of ca. 1.6. For comparison, the investigations conducted in one of the composting plants in China revealed the summary HI parameter value in the range from 0.5 to 3.18, depending on location and season of the year [16]. Nevertheless, the results of our study show that special attention should be paid to the problem of volatile odorous compounds emission, particularly in the locations responsible for mechanical sewage treatment and composting process.

**Figure 5:**
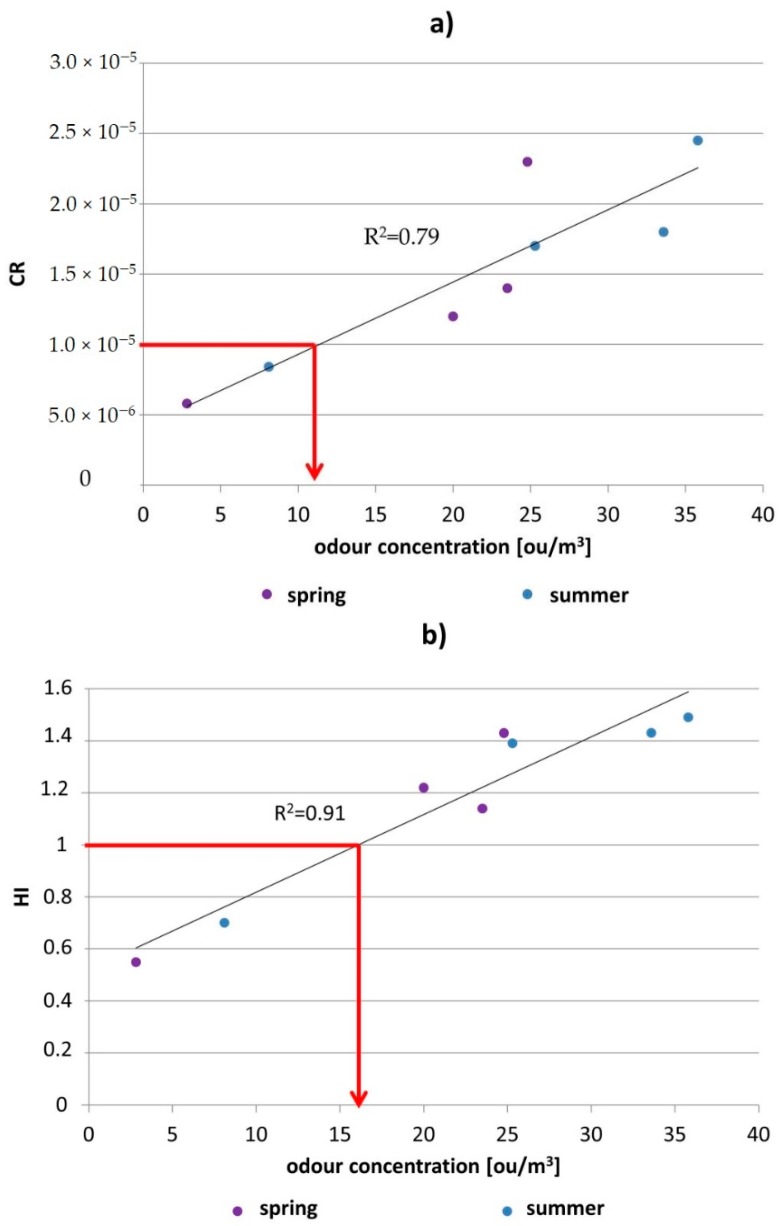
Dependence between CR and HI parameters and odour concentration: (**a**) CR, (**b**) HI.

Figure 4a,b also contain the odour concentrations in given measurement locations. The highest c_od_ values were estimated for SWCP (37.2 ou/m^3^ in the summer season of 2018, 24.9 ou/m^3^ in the spring season of 2018). The lowest values occurred for the biological reactors area (8.11 ou/m^3^ during the summer season of 2017 and 3.73 ou/m^3^ during the spring season—both 2017 and 2018). It seems justified to conclude that odour concentrations are correlated with the values of CR and HI parameters; the higher the odour concentrations in a vicinity of the measurement sites, the higher the obtained values of the CR and HI parameters. Comparison of the results illustrated in Figure 4a shows that if odour concentration is above 10 ou/m^3^, the CR parameter exceeds “probable risks” level (Figure 5a). Comparing the results presented in Figure 4b, it can also be qualitatively stated that if the odour concentration is above 15 ou/m^3^, the HI parameter values will exceed the acceptable level (Figure 5b). In some cases such a situation would make it possible in the future to employ only olfactometric investigations to health risk assessment. Moreover, the olfactometric studies would lower the cost of continuous air quality monitoring in wastewater treatment plants. Obviously, it will require additional, many years’ instrumental and olfactometric investigations to confirm this hypothesis.

The results of performed investigations indicate that the potential phenomenon of odour nuisance, due to the wastewater treatment plant operation, results mainly from increased emission of malodorous substances during mechanical sewage treatment as well as composting of sludge and solid waste from the mechanical treatment section. Similar observations are reported in the literature concerning the occurrence of odours in wastewater treatment plants. Lebrero et al. [30] showed that the biggest contribution to potential odour nuisance of a wastewater treatment plant originated from the operations of preliminary treatment and processing of sludge (thickening, dewatering, anaerobic fermentation). Capelli et al. [34] compared odour concentration levels in 17 different wastewater treatment plants, taking into account 10 potential emission sources. Investigated treatment plants were divided into three groups regarding their size (“small”—treatment capacity within 10^3^–10^4^ m^3^ per day, “medium”—within 10^4^–10^5^ m^3^ per day and ‘‘large’’ within 10^5^–10^6^ m^3^ per day). The highest odour concentrations were estimated in a stream of new-supplied sewage, during their mechanical treatment and during the operations connected with thickening and dewatering of sludge (odour concentrations amounted ca. 1000 ou_E_/m^3^). Based on the investigation, the authors concluded that the size of the plants did not have a significant impact on the magnitude of emission, both in particular sources as well as on the main source of malodorous compounds.

## 4. Conclusions

In this paper the authors presented a human health risk assessment for one of the wastewater treatment plants in northern Poland, according to the guidelines of the US EPA and the information contained in the IARC documents, taking into account the odour concentrations evaluated at the air samples collection sites. The investigations revealed that when analysing only the CR and HI parameters for particular compounds from the VOCs group, only in a minority of cases the substances present in selected measurement locations could influence the deterioration of the health of the plant’s employees and the residents of the neighbouring areas. The highest health risk to the plant’s workers originates from the substances, which are considered carcinogenic according to the IARC guidelines. Based on the CR parameters determined for these compounds following the US EPA methodology, it can be stated with certain probability that these substances, especially formaldehyde, may cause cancer disorders among the plant’s employees. However, analysis of the summary values of the CR and HI parameters shows that, in selected locations, the HI parameter exceeds the accepted level, and the CR parameter is above the “probable risks” level. Moreover, it was observed that the odour concentrations were relatively well-correlated with the CR and HI parameters values; the odour concentrations above 10 ou/m^3^ corresponded to the CR parameter at the level of 1.0 × 10^−5^ (R^2^ = 0.79) and the odour concentartion above 15 ou/m^3^ was connected with the HI parameter higher than 1 (R^2^ = 0.91). Hypothetically, such a situation allows for the application of only olfactometric measurements to health risk assessment regarding carcinogenity. In the future, it would be enough to control the odour concentration level with the olfactomtric techniques and exceedence of accepted CR and HI parameters limits would be confirmed instrumentally. Such a situation would contribute to lower analysis cost and to a decrease in labour consumption. Obviously, confirmation of this hypothesis calls for further instrumental and olfactometric studies as well as toxicological examinations of the workers exposed to VOCs emission from the wastewater treatment plant.

The investigations showed that the health risk assessment of the wastweater treatment plant workers conducted with the instrumental and sensory methods was justified. Summary values of the CR and HI parameters indicate that new or improved solutions regarding the reduction of odour emission from the wastewater treatment plant should be implemented. Relatively high odour concentrations occuring in the volatile organic compounds emission sites also influence work discomfort in the plant.

## Figures and Tables

**Table 1:** Information about the amount of processed wastewater and sludge during the investigation period.

WWTPs Characteristics	2017	2018
Amount of wastewater supplied to the WWTP (m^3^/day)	14,500	15,200
Amount of wastewater discharged from the WWTP (m^3^/day)	8479	9360
Amount of generated sludge (t/year)	7207	8076

**Table 2:** Average percentage contribution of chemical classes at each area in WWTP under investigation (HMT—hall of mechanical treatment, BRA—biological reactors area, SCP—sludge composting piles, SWCP—solid waste composting piles).

Percentage Contribution of Chemical Classes (%)								
Chemical Classes	HMT		BRA		SCP		SWCP	
	Summer	Spring	Summer	Spring	Summer	Spring	Summer	Spring
Sulphur compounds	27.1 ± 1.6	22.9 ± 1.6	7.7 ± 0.3	7.9 ± 0.2	29.1 ± 1.3	29.9 ± 1.1	31.4 ± 1.4	30.8 ± 2.0
Aromatic hydrocarbons	20.8 ± 1.4	19.7 ± 1.3	11.9 ± 0.4	12.9 ± 0.5	16.7 ± 1.0	12.9 ± 0.6	13.8 ± 0.6	15.0 ± 0.5
Aldehydes	11.2 ± 0.7	8.6 ± 0.6	14.2 ± 0.6	11.4 ± 0.5	8.2 ± 0.4	10.1 ± 0.5	9.2 ± 0.2	7.4 ± 0.3
Ketones	6.3 ± 0.3	6.7 ± 0.2	20.2 ± 0.9	22.4 ± 0.3	8.1 ± 0.5	9.4 ± 0.4	11.4 ± 0.4	10.4 ± 0.6
Aliphatic hydrocarbons	11.6 ± 0.6	12.7 ± 0.6	24.8 ± 1.2	23.1 ± 0.5	14.7 ± 0.8	15.6 ± 0.7	8.8 ± 0.4	9.9 ± 0.7
Alcohols	13.8 ± 0.8	16.9 ± 0.9	15.4 ± 1.1	21.1 ± 0.9	12.8 ± 1.1	12.0 ± 0.7	19.1 ± 0.6	19.7 ± 0.9
Others	9.2 ± 0.5	12.5 ± 0.4	5.8 ± 0.7	1.2 ± 0.2	10.4 ± 0.6	10.1 ± 0.7	6.3 ± 0.7	6.8 ± 0.7

**Table 3:** Chemical compounds identified in this study (olfactory threshold values from [31]; n.d.—no data).

1RT (s)	2RT (s)	Name of Compounds	Similarity	Odour Descriptors	Olfactory Threshold (ppm)
380	2.18	n-pentane	932	mild, sweet, gasoline	1.4
410	1.98	n-hexane	895	mild, gasoline-like	1.5
530	2.01	n-heptane	938	decayed, cabbage	6.7 × 10^−1^
935	2.06	n-nonane	802	sharp	2.2
465	2.17	benzene	886	gasoline-like	2.7
925	2.35	ethylbenzene	931	aromatic	1.7 × 10^−1^
876	2.11	propyl benzene	910	n.d.	3.8 × 10^−3^
640	2.30	toluene	873	paint, solvent	3.3 × 10^−1^
895	2.39	o-xylene	872	sweet, rubber	3.8 × 10^−1^
845	2.32	m-xylene	927	sweet	4.1 × 10^−2^
910	2.49	p-xylene	907	sweet	5.8 × 10^−2^
1135	2.42	1,3,5-trimethylbenzene	905	sweet, aromatic	1.7 × 10^−1^
1210	2.11	o-cresol	913	phenol-like	2.8 × 10^−4^
1214	1.99	m-cresol	955	sweet tarry, phenol-like	1.0 × 10^−4^
1220	2.32	p-cresol	833	feces	5.4 × 10^−5^
1090	1.58	phenol	935	sweet tarry, acrid, sweet	5.6 × 10^−3^
330	2.09	acetone	922	sweet, solvent	4.2 × 10^1^
485	2.11	methyl ethyl ketone	918	pleasant pungent	4.4 × 10^−1^
547	2.63	methyl isobutyl ketone	938	pleasant, camphor	5.0 × 10^−1^
622	1.89	2-hexanone	944	pungent, acetone-like	2.4 × 10^−2^
695	2.30	heptanal	882	pungent, linen	1.8 × 10^−4^
620	1.98	formaldehyde	916	pungent, irritating	5.0 × 10^−1^
745	2.28	acetaldehyde	922	ethereal	1.5 × 10^−3^
425	2.32	methyl tert-butyl ether	901	distinctive, anesthetic-like	n.d.

1RT—first retention time; 2RT—second retention time; similarity—fitting probability between the mass spectrum obtained via chromatographic analysis and the spectrum contained in the NIST 2011 spectra library. Application of this algorithm allows for identification of measured substances.

**Table 4:** Comparison of CR and HI values obtained in this study with other studies.

Study	CR	HI	Ref.
This study	1.4 × 10^−7^–1.6 × 10^−5^	7.0 × 10^−5^–6.0 × 10^−1^	-
BTEX emission in landfill environment	6.7 × 10^−5^	1.4 × 10^−2^–1.9 × 10^−1^	[19]
VOCs emisssion in landfill area	3.5 × 10^−11^–8.8 × 10^−6^	2.6 × 10^−4^–4.4 × 10^−1^	[33]
	9.7 × 10^−7^–1.3 × 10^−4^	6.7 × 10^−5^–7.3 × 10^−1^	[32]
VOCs emission in composting plant	1.8 × 10^−8^–1.1 × 10^−4^	3.3 × 10^−6^–0.3 × 10^1^	[20]
	5.5 × 10^−7^–1.9 × 10^−4^	7.6 × 10^−4^–4.0 × 10^−1^	[21]

**Table 5:** Comparison of average concentration of identified compounds emitted during sewage treatment with acceptable concentration levels. Classification of these acompounds according to US EPA and International Agency for Research on Cancer (IARC).

Name of Compound	Average Concentration (µg/m^3^)	Acceptable Concentration Averaged over One Calendar Year (µg/m^3^)	Classification According to US EPA	Classification According to IARC
n-pentane	13.67	1000	HI	-
n-hexane	6.99	1000	HI	-
n-heptane	7.86	1000	HI	-
n-nonane	4.66	1000	HI	-
benzene	3.03	5	CR, HI	1
ethylbenzene	4.83	38	CR, HI	2B
propyl benzene	4.61	13	HI	-
toluene	40.49	10	HI	3
o-xylene	8.88	10	HI	3
m-xylene	12.65	10	HI	3
p-xylene	18.00	10	HI	3
1,3,5-trimethylbenzene	3.24	-	HI	-
o-cresol	6.11	1.6	HI	-
m-cresol	7.50	1.6	HI	-
p-cresol	10.04	1.6	HI	-
phenol	18.29	2.5	HI	3
acetone	17.07	30	HI	-
methyl ethyl ketone	8.29	26	HI	-
methyl isobutyl ketone	9.00	3.8	HI	2B
2-hexanone	4.16	-	HI	-
heptanal	3.40	-	HI	-
formaldehyde	6.00	4	CR, HI	1
acetaldehyde	5.63	2.5	CR, HI	2B
methyl tert-butyl ether	6.79	-	CR, HI	3
